# Flow Cytometric Assessment of Bacterial Abundance in Soils, Sediments and Sludge

**DOI:** 10.3389/fmicb.2016.00903

**Published:** 2016-06-14

**Authors:** Aline Frossard, Frederik Hammes, Mark O. Gessner

**Affiliations:** ^1^Forest Soils and Biogeochemistry, Swiss Federal Institute for Forest, Snow and Landscape Research (WSL)Birmensdorf Switzerland; ^2^Department of Aquatic Ecology, Swiss Federal Institute of Aquatic Science and Technology (Eawag)Dübendorf Switzerland; ^3^Department of Experimental Limnology, Leibniz Institute of Freshwater Ecology and Inland Fisheries (IGB), StechlinGermany; ^4^Institute of Integrative Biology (IBZ), ETH ZürichZürich Switzerland; ^5^Department of Environmental Microbiology, Swiss Federal Institute of Aquatic Science and Technology (Eawag)Dübendorf Switzerland; ^6^Department of Ecology, Berlin Institute of Technology (TU Berlin)Berlin Germany

**Keywords:** flow cytometry, epifluorescence microscopy, ATP, bacterial abundance, sediment, soil, drinking water treatment plant sand-filters

## Abstract

Bacterial abundance is a fundamental measure in microbiology, but its assessment is often tedious, especially for soil, and sediment samples. To overcome this limitation, we adopted a time-efficient flow-cytometric (FCM) counting method involving cell detachment and separation from matrix particles by centrifugation in tubes receiving sample suspensions and Histodenz^®^ solution. We used this approach to assess bacterial abundances in diverse soils (natural and agricultural), sediments (streams and lakes) and sludge from sand-filters in a drinking water treatment plant and compared the results to bacterial abundances determined by two established methods, epifluorescence microscopy (EM) and adenosine triphosphate (ATP) quantification. Cell abundances determined by FCM and EM correlated fairly well, although absolute cell abundances were generally lower when determined by FCM. FCM also showed significant relations with cell counts converted from ATP concentrations, although estimates derived from ATP determinations were typically higher, indicating the presence of ATP sources other than bacteria. Soil and sediment organic matter (OM) content influenced the goodness of fit between counts obtained with EM and FCM. In particular, bacterial abundance determined by FCM in samples containing less than 10% OM, such as stream sediment, was particularly well correlated with the cell counts assessed by EM. Overall, these results suggest that FCM following cell detachment and purification is a useful approach to increase sample throughput for determining bacterial abundances in soils, sediments and sludge. However, notable scatter and only partial concordance among the FCM and reference methods suggests that protocols require further improvement for assessments requiring high precision, especially when OM contents in samples are high.

## Introduction

Heterotrophic prokaryotes are a dominant component of the biosphere. They recycle an extremely large fraction of organic matter (OM) in most biomes and reach an estimated total of 4–6 × 10^30^ cells on earth (Whitman et al., 1998). Discovered more than three centuries ago, prokaryotes (including *Bacteria* and *Archaea*) were initially studied primarily by direct microscopic observations. Subsequently, colonies derived from single cells were counted using plating techniques to assess bacterial abundance. However, it has been established for decades that the great majority of prokaryotes fails to grow on culture media, and this situation has not changed despite considerable progress in culturing techniques (Alain and Querellou, 2009). This has prompted the use and further development of culture-independent techniques to examine microbes in the environment (Amann et al., 1995; Pham and Kim, 2012). One of the most basic variables to determine in both natural and technical systems is bacterial abundance. Its assessment, however, remains tedious for many types of samples. Therefore, there is an urgent need for rapid and reliable techniques to estimate bacterial cell numbers (and biomass) in diverse environments, a requirement that has long been identified as a priority (Davis, 2014).

Direct microscopic counts of bacterial cells stained with fluorescent dyes, such as acridine orange (AO) and 4′,6-diamidino-2-phenylindole (DAPI), have been widely applied to environmental samples from the early 80’s onward (Kepner and Pratt, 1994). Application of these methods boosted quantitative analyses of bacterial and archaeal cells in natural environments by overcoming the previous strong biases arising from plating methods. Being a straightforward method, direct counts of stained cells thus opened new perspectives to study bacteria and archaea in natural environments (Cragg and Parkes, 2014). For example, estimates of total bacterial abundance became instrumental in developing the microbial loop concept for marine plankton (Azam et al., 1983), which was later extended to lakes (Weisse et al., 1990) and, to some extent, also to other environments such as soils (Clarholm and Rosswall, 1980; Bonde et al., 1988), streams (Meyer, 1994) and technical systems (Prevost et al., 1998). Over the years, a wide variety of improved fluorescent dyes have been proposed and successfully employed for direct microscopic counts, including dyes of the SYBR Green family originally developed for staining nucleic acids separated on gels (Schneeberger et al., 1995). Later, the approach was also extended to enumerate flagellates (Marie et al., 1997) and free-living viruses (Noble and Fuhrman, 1999) in lakes and oceans.

Although a major methodological breakthrough at the time that led to fundamental insights and conceptual advances, direct counts by epifluorescence microscopy (EM) suffer from two major limitations: (1) They involve considerable observer bias, requiring strictly standardized counting procedures and thorough cross-calibration among individuals to ensure reproducible results, and (2) they are notoriously time-consuming. These shortcomings led environmental microbiologists to seek alternative approaches to quantify bacteria in the environment, including semi-automated counts (Pernthaler et al., 2003), real-time PCR (qPCR; Fierer et al., 2007), and adenosine tri-phosphate (ATP) analyses (Karl, 1980; Hammes et al., 2010). One of the most promising alternatives consists of counting cells by flow cytometry (FCM) following staining of nucleic acid-containing particles with some of the same fluorescent dyes used for direct microscopic cell counts. FCM overcomes both of the main disadvantages of epifluorescent microscopy, namely observer bias and low sample throughput, facilitating the analysis of much larger sample sizes compared to microscopic methods, and hence increasing reliability of cell abundance estimates, while tremendously reducing sample processing time. As a result, flow cytometric cell counts are becoming increasingly established as a routine method for a variety of applications (Hammes and Egli, 2010), even though information on cell size and shape, which can be obtained by epifluorescent microscopy, is largely lost when using flow cytometry.

Use of flow cytometry in microbiology was originally restricted to cell quantification of pure cultures (Czechowska et al., 2008), since inorganic particles can strongly interfere with bacterial counts. Therefore, although now widely applied to water samples from fresh waters and oceans, reliable flow cytometric enumeration of microbial cells associated with surfaces in sediments, soils and other environments remains challenging, requiring detachment and mechanical or optical separation of cells from interfering particles. Several protocols for separating cells from the soil and sediment matrix have been proposed and tested (Epstein et al., 1997; Buesing and Gessner, 2002; Maron et al., 2006; Kallmeyer et al., 2008). They involve chemical (e.g., by ionic and non-ionic detergents such as Tween and sodium pyrophosphate, respectively), mechanical (by ultrasonication or blenders; Falcioni et al., 2006) or enzymatic treatments (Böckelmann et al., 2003), or a combination of these (Amalfitano and Fazi, 2008). Cell detachment using ultrasonication (Buesing and Gessner, 2002) and separation by density-gradient centrifugation (Amalfitano and Fazi, 2008; Morono et al., 2013) are particularly promising to prepare samples for flow-cytometric enumeration (Amalfitano et al., 2009). However, no systematic comparisons of the efficacy of this approach across a range of distinct environmental samples are currently available.

The main objective of this study was to test the reliability of flow cytometry for bacterial cell counts in soil, sediment and sludge samples by comparing this method with two other well established methods, namely EM and ATP analysis. Bacterial abundance was assessed using these three methods in parallel with samples collected from a wide variety of aquatic and terrestrial environments, including natural and agricultural soils, stream and lake sediments, and sludge from sand filters of a drinking water treatment plant.

## Materials and Methods

### Sample Collection and Preparation

Samples were collected between June 2009 and April 2011 from streams (12) and lake sediments (15), the surface sludge layer of slow sand filters in a drinking water treatment plant (8) and soils of both natural (12) and agroecosystems (8) (Supplementary Table S1). Within 24 h after collection, subsamples of ca. 3 g wet mass were transferred to 20-ml sterilized glass vials containing either 10 ml of a 2% paraformaldehyde (PFA) solution (buffered with 0.1% sodium pyrophosphate) for analyses by EM and flow cytometry, or 10 ml of phosphate-buffered saline (PBS) (130 mM NaCl, 7 mM Na_2_HPO_4_, 3 mM NaH_2_PO_4_) for ATP analysis. All vials were stored at 4°C in the dark until analyzed. Bacterial cells were detached from soil, sediment or sludge by treatment for 1 min (3 × 20 s with breaks of 20 s in between) with an ultrasonic probe (Branson Digital Sonifier 250, Danbury, CT, USA) with an actual output of 38 W at the flat tip (for details see Buesing and Gessner, 2002). The resulting bacterial cell suspension was homogenized by vortexing and a 1-ml subsample was placed on top of 0.5 ml of Histodenz^®^ solution (1.3 g ml^-1^, Sigma–Aldrich, Buchs, Switzerland) in a sterile reaction tube. The tubes were then centrifuged (90 min at 4°C and 17,135 ×*g*) and the entire upper layer was kept while the underlying Histodenz^®^ layer was discarded.

All soil, sediment and sludge samples were dried at 105°C to constant mass and combusted in a muﬄe furnace (4 h at 450°C) to determine total dry mass and OM content.

### Flow Cytometry

Bacterial cells in the cleaned suspensions were stained with SYBR Green I in anhydrous dimethylsulfoxide (DMSO) and incubated in the dark for 15 min. The samples were then diluted 1:10 or 1:100 with filtered (0.22 μm Millex^®^-GP, Millipore, Wohlen, Switzerland) mineral water (Evian, France) such that the cell concentration did not exceed 10^6^ ml^-1^. Samples were analyzed with a CyFlow^®^ space Flow Cytometer System (Partec, Görlitz, Germany) equipped with a 200 mW solid-state laser emitting light at 488 nm. Green and red fluorescence were measured at 520 nm (FL1 channel) and 630 nm (FL3 channel). The flow cytometer was set as follows: gain FL1 = 495, gain FL3 = 50, speed = 4 (implying an event rate never exceeding 1000 events per second). Counts were recorded as logarithmic signals and were triggered on the green fluorescence channel (FL1). Data were processed with Flowmax software (Partec, Görlitz, Germany), using electronic gating to separate the desired events. Presentation of the data as FL1/FL3 dot plots allowed for optimal distinction between stained intact microbial cells and instrument noise or sample background (Hammes and Egli, 2005).

### Epifluorescence Microscopy

Abundance and biomass of detached bacterial suspension was determined by epifluorescence microscopy of samples stained with SYBR Green I (Buesing, 2005). Bacterial cell suspensions were diluted 50 times and vortexed for 30 s. Aliquots (30–500 μl) were placed into a vacuum filtering-manifold containing 3 ml of sterile nanopure water. An additional 3 ml of sterile water was added to ensure a homogenous distribution of bacterial cells in the suspension. Samples were gently filtered (vacuum ≤200 mbar) on an Anodisc filter (0.2 μm pore size, 25 mm diameter, Whatman, Bottmingen, Switzerland) placed on top of a backing cellulose filter (0.45 μm pore size, Millipore, Zug, Switzerland). Filters were dried for 15 min and subsequently stained for 15 min by placing the filter on a drop of 100 μl of SYBR Green solution (0.25%), and rinsing with 3 ml of sterile nanopure water. Finally, the filters were dried in the dark for 15 min and placed on a microscope slide. Before covering the filter with a cover slip, 30 μl of antifading solution (50% of 87% glycerol, 50% of PBS, and 0.1% of 5% *p*-phenylenediamine) was evenly distributed on the filter. The slides were observed under an epifluorescence miscroscope (Leica Microsystem DMI6000 B, GFP filter, gain = 5, intensity = 3, brightness = 20) at 1000 × magnification using oil immersion (Leica, Heerbrugg, Switzerland). Pictures of at least 20 microscopic fields per filter were taken so that a total of ≥400 cells per filter were counted (Norland et al., 1987). Pictures were analyzed with image analysis software ImageJ (Schneider et al., 2012).

### ATP

Total ATP concentration was determined with the BacTiter-Glo reagent (Promega Corporation, Madison, WI, USA) along with a luminometer (Glomax, Turner Biosystems, Sunnyvale, CA, USA) as described in Hammes et al. (2010). Briefly, samples stored in PBS buffer within 24 h after sample collection were sonified as described above before 500-μl aliquots of the suspension were diluted with sterile nanopure water (100 to 10000×) and transferred into a 2-ml sterile reaction tube that was heated for at least 10 min in a heating block (38°C). ATP reagent was transferred to a separate sterile reaction tube and heated for at least 1 min. Samples were then transferred to the reagent tube and the mixture was incubated for another 20 s. Luminescence was subsequently measured as an integral over 10 s, expressed in relative light units (RLU). RLU were converted to ATP concentration based on a calibration curve established with pure ATP standard (Promega, Madison, WI, USA) over a concentration range of 10^-6^ to 10^-1^ μg L^-1^ of ATP. A conversion factor of 8.9 × 10^-17^ g ATP per cell was used to calculate cell abundance (Hammes et al., 2010).

### Data Analysis

Linear models were used to detect significant differences among the three methods used to assess bacterial abundance (i.e., FCM, EM, and ATP; function *lm*, R Development Core Team, 2014). Since QQ-plots and frequency histograms indicated that residuals did not meet the assumptions required for parametric tests, variables were log_10_-transformed prior to analyses. Orthogonal regressions were then calculated between log-transformed cell abundance assessed by FCM and either EM or ATP. Coefficients of determination (*r*^2^) and significance levels (P) were used to test for the strength of relationships between the three methods for the whole dataset and for data obtained separately from each environment and for different OM contents.

## Results and Discussion

**FIGURE 1:**
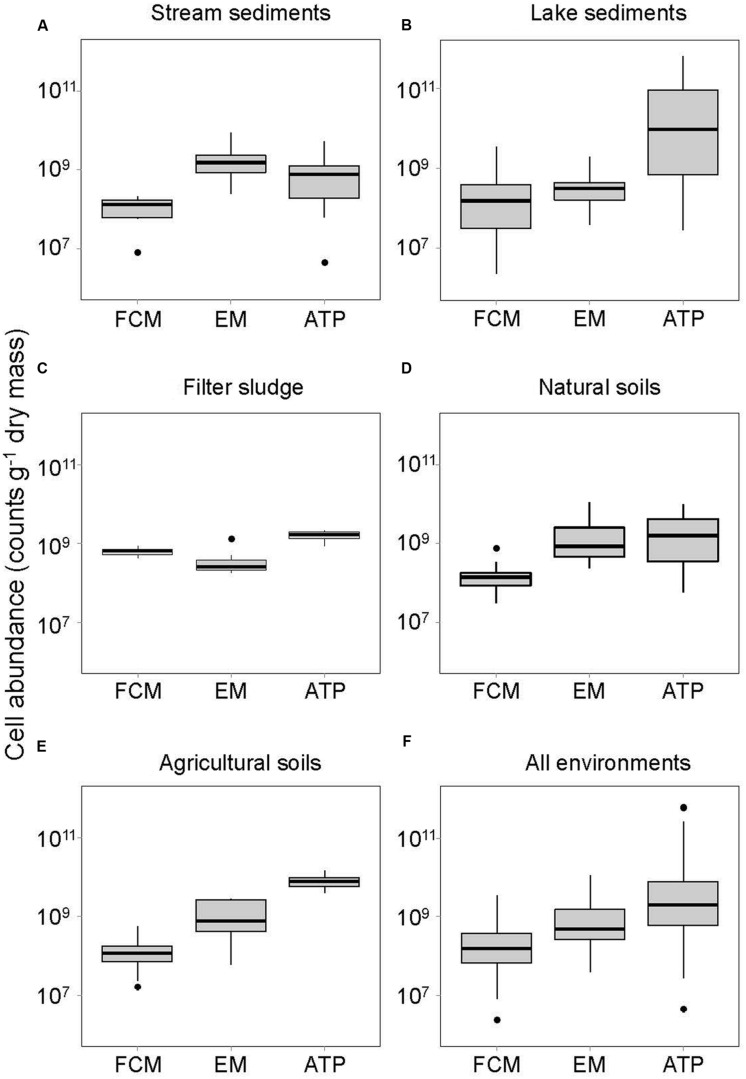
**Bacterial abundances assessed by three different methods (FCM, flow cytometry; EM, epifluorescence microscopy; ATP, ATP quantification) in (A) stream sediments, (B) lake sediments, (C) natural soils, (D) agricultural soils, (E) filter sludge of a drinking water treatment plant, and (F) all environments together**. Horizontal thick lines show median values, boxes denote values comprised within the lower and upper quartile of the data, thin vertical lines represent ranges, and • indicate outliers.

Bacterial cells numbers in the contrasting soil, sediment and sludge environments of streams and lakes, natural and agricultural soils as well as sand filters in a drinking water treatment plant varied with the method used to assess bacterial abundance (**Table 1**; **Figure 1**). FCM yielded significantly lower bacterial abundances than EM in most environments (Supplementaey Table S2). Only the sludge of sand filters had higher bacterial numbers when counted by FCM. Cell numbers determined by FCM averaged 3.4 × 10^8^± 5.6 × 10^8^ g^-1^ DM, whereas an average of 1.4 × 10^9^± 2.3 × 10^9^ cells g^-1^ DM was obtained by EM, and an average of 3.3 × 10^10^± 1.2 × 10^11^ cells g^-1^ DM when cell number estimates were based on ATP analyses (**Table 1**; **Figure 1**). These data contrast with results from a study in marine sediments where more bacterial cells were detected by FCM than by EM (Lavergne et al., 2014). However, the cells counted by EM in that study were stained with DAPI, whereas in the present study, the same dye, SYBR Green I, was used for both FCM and EM. However, the binding efficiency and affinity to DNA and RNA varies considerably among dyes, with SYBR Green stains being more effective in binding specifically to DNA and RNA than DAPI. Therefore, if DAPI staining led to unspecific binding of the dye to non-bacterial particles and thus an overestimation of bacterial numbers, the comparison of cell numbers in the study by Lavergne et al. (2014) might have been biased by the use of different dyes (Marie et al., 1997; Troussellier et al., 1999). Alternatively, it is possible that FCM led to an underestimation of bacterial numbers, although an obvious reason why counting by FCM could have been inefficient is not apparent.

**Table 1:** Bacterial abundance in different environments expressed as cells g^-1^ of sediment, soil, or sludge dry mass assessed by three different methods (FCM, flow cytometry; EM, epifluorescence microscopy; ATP, ATP quantification).

Environment	*N*	%OM	FCM		EM		ATP	
			Mean	*SD*	Mean	*SD*	Mean	*SD*
All environments	55	0.01–43.6	3.4 × 10^8^	5.6 × 10^8^	1.4 × 10^9^	2.3 × 10^9^	3.3 × 10^10^	1.2 × 10^11^
Stream sediments	12	0.01-2.61	1.2 × 10^8^	6.5 × 10^7^	2.5 × 10^8^	1.5 × 10^8^	1.3 × 10^9^	1.7 × 10^9^
Lake sediments	15	2.29-36.9	5.6 × 10^8^	10.0 × 10^8^	2.5 × 10^9^	3.1 × 10^9^	1.1 × 10^11^	2.1 × 10^11^
Filter Sludge	8	0.51-0.72	6.2 × 10^8^	1.5 × 10^8^	4.8 × 10^8^	2.6 × 10^8^	1.6 × 10^9^	4.5 × 10^8^
Natural soils	12	0.09-7.75	1.9 × 10^8^	2.0 × 10^8^	5.9 × 10^8^	5.8 × 10^8^	3.0 × 10^9^	3.5 × 10^9^
Agricultural soils	8	2.77-43.6	1.8 × 10^8^	1.8 × 10^8^	3.4 × 10^9^	2.9 × 10^9^	7.9 × 10^9^	3.3 × 10^9^

*Ranges of organic matter (OM) content (%) are indicated for each environment. Bacterial abundances derived from ATP concentrations are based on a conversion factor of 8.9 × 10^-*17*^ g ATP per cell (Hammes et al., 2010)*.

**FIGURE 2:**
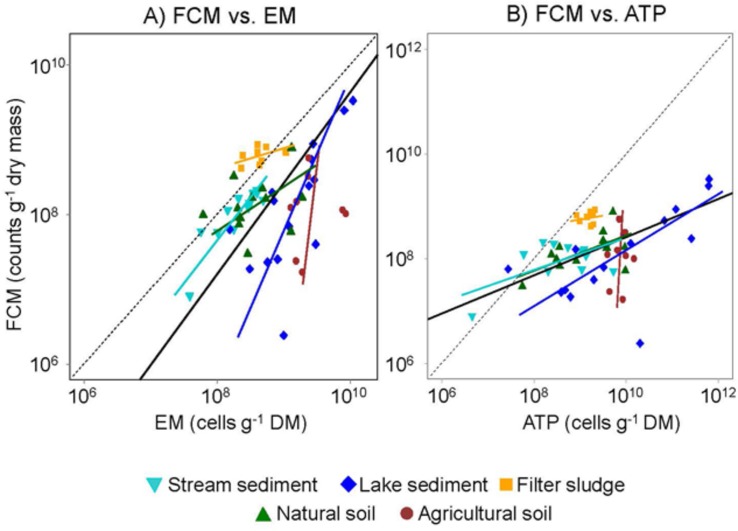
**Relationships between bacterial abundances assessed by FCM and either (A) EM or (B) ATP**. Colors and shape represent five different types of environments that were sampled. Slopes of orthogonal regression lines for each environment are indicated with the same color and shape code: (1) stream sediment, (2) Lake sediment, (3) Filter sludge, (4) Natural soils, and (5) Agricultural soils. Solid black lines indicate the overall regression lines. Black dashed lines represents the 1:1 line, i.e., the theoretical ideal relationship between estimates obtained with the different methods.

Although the question currently remains open whether the difference between bacterial abundances detected in our study was due to an overestimation by EM or an underestimation by FCM, the abundances obtained with both methods were significantly related to the ATP concentration when the data are pooled across all environments (*P* < 0.001, *r*^2^= 0.19 and 0.60 for FCM and EM, respectively; **Figure 2**). This outcome may seem surprising in view of numerous studies reporting that about 40% of freshwater and 80% of soil microbial biomass is considered dormant (Lennon and Jones, 2011). However, although greatly reduced in inactive cells, ATP is still needed for maintenance metabolism (Van Bodegom, 2007). Consequently, a fraction of the biomass detected by the ATP assay could be accounted for by dormant bacterial cells (Blagodatskaya and Kuzyakov, 2013). Furthermore, ATP in other microorganisms, such as fungi, or in micro-invertebrates as well as fine roots and small plant residues was present in the samples and hence likely to contribute to the total ATP pool.

For all environments except stream sediments, estimates based on the ATP assay yielded significantly higher average bacterial abundances than the FCM method (**Table 1**; **Figure 1**, Supplementary Table S2). However, cell abundances derived from the ATP assay also showed greater variation than the cell numbers obtained by FCM (**Figure 1**). Differences in the preservation method (PFA for FCM, no preservative for ATP analyses), might partly account for this discrepancy. Although PFA is a standard preservative for storing environmental samples for bacterial counts, a decline in bacterial cells has been reported from formaldehyde-preserved samples, including from lake sediments (Duhamel and Jacquet, 2006). As samples for EM and FCM analyses were stored in formalin for 1–4 weeks after collection (depending on the sample type) compared to 24 h maximum in a simple buffer for ATP, we cannot rule that cell numbers assessed by EM and FCM were underestimated. However, this would not affect the comparison of these two methods.

Despite differences in absolute numbers, the cell abundances assessed by FCM were significantly related to those determined by EM when data from all environments are combined (*P* = 0.02, *r*^2^ = 0.10; **Figure 2**; **Table 2**). However, when the data was analyzed for each environment separately, tight relations between FCM and EM were only observed for stream and lake sediments (*P* < 0.001 and 0.004, *r*^2^ = 0.77 and 0.48, respectively), environments where the greatest differences in cell number among samples were found. Moreover, bacterial abundances in stream sediments showed an orthogonal regression slope between FCM and EM close to the theoretical slope of 1:1 for an almost perfect fit between the two methods (slope = 1.14; **Table 2**; **Figure 2**). Provided that EM is an appropriate reference method, this result suggests that the coarse stream sediments examined in our study, more than the other environments tested, are well suited to assess bacterial abundance by FCM. Based on an analysis of river sediment samples, Amalfitano et al. (2009) drew a similar conclusion. Since cell numbers in filter sludge varied much less among samples, one would not expect a strong relation between FCM and EM counts. It is notable, however, that all data points are close to the 1:1 line (**Figure 2A**), suggesting that both methods resulted in very similar estimates as well.

**Table 2:** Slopes, intercepts, coefficients of determination (*r*^2^) and significance levels (P) of orthogonal regressions of log-transformed cell abundances g^-1^ dry mass estimated by FCM, EM, and ATP assays of samples from five different types of environments.

Environment	*N*	FCM vs. EM				FCM vs. ATP			
		Slope	Intercept	*r*^2^	*P*	Slope	Intercept	*r*^2^	*P*
All environments	55	1.22	-2.6	0.10	0.020^∗^	0.36	4.8	0.19	0.001^∗^
Stream sediments	12	1.14	-1.5	0.77	<0.001^∗^	0.31	5.3	0.32	0.054
Lake sediments	15	2.07	-10.8	0.48	0.004^∗^	0.54	2.8	0.44	0.007^∗^
Filter sludge	8	0.27	6.4	0.17	0.312	0.13	7.6	<0.01	0.866
Natural soils	12	0.59	3.1	0.08	0.367	0.34	5.1	0.31	0.058
Agricultural soils	8	7.51	-63	0.02	0.750	15	-142	0.02	0.709

*^∗^*p* < 0.05*.

Organic matter content in sediment, soil and sludge samples appeared to influence the relationship between FCM and EM counts. Specifically, samples containing less than 10% of OM showed a significant relationship between FCM and EM counts (**Table 3**). Moreover, when only data from samples with an OM content of 10% or less were included in the analysis, the slope of the relationship between FCM and EM counts was close to the theoretical slope of 1 (**Table 3**).

**Table 3:** Slopes, intercepts, coefficients of determination (*r*^2^) and significance levels (P) of orthogonal regressions of log-transformed cell abundances g^-1^ dry mass estimated by FCM, EM, and ATP assays for data sets including increasingly lower OM contents between <45% (includes all samples) and <1%.

Organic matter content (%)	*N*	FCM vs. EM				FCM vs. ATP			
		Slope	Intercept	*r*^2^	*P*	Slope	Intercept	*r*^2^	*P*
<45	55	1.22	-2.6	0.10	0.020^∗^	0.37	4.8	0.19	0.001^∗^
<20	49	2.23	-11.3	0.05	0.107	0.35	5.0	0.10	0.029^∗^
<10	47	1.24	-2.5	0.12	0.019^∗^	0.37	4.8	0.21	0.001^∗^
<5	39	1.28	-2.8	0.15	0.013^∗^	0.42	4.5	0.23	0.002^∗^
<2	24	1.71	-6.1	0.53	<0.001^∗^	0.65	2.6	0.47	<0.001^∗^
<1	19	1.75	-6.5	0.56	<0.001^∗^	0.77	1.6	0.74	<0.001^∗^

*^∗^*p* < 0.05*.

Although the density gradient centrifugation included in the sample preparation of our protocol removed most OM, as also found by Amalfitano and Fazi (2008) and Poté et al. (2010), some of the inorganic and organic particles can remain in the cell fraction after centrifugation (personal observation). Inorganic particles should not be stained by SYBR Green, which binds specifically to nucleic acids, but viral particles (Noble and Fuhrman, 1998) and extracellular DNA (Soler et al., 2008) are stained and could possibly contribute to false positives, especially when adhering to bacterial-sized mineral or dead organic particles. Therefore, when detected by flow cytometry, these particles might reduce the accuracy of the technique, making it difficult to distinguish cells from interfering particles, and thus bias the cell counts. However, similar biases also complicate the EM method, which might in fact explain the greater cell numbers observed by EM compared to the FCM method (**Figure 1**) in all environments but the stream sediments. Moreover, although cell shape can be evaluated under the epifluorescence microscope, the cells and interfering particles were visually differentiated, potentially increasing error likelihood, whereas the FCM technique can be better standardized through fixed gating (Prest et al., 2013). Therefore, the precision of the FCM method is likely to be higher than that of the microscopic method. Accordingly, Wang et al. (2010) found the standard deviation between replicate samples to be <5% for FCM but>10% for EM.

## Conclusion

Flow-cytometric assessment of bacterial abundance is now routinely used for water samples (Wang et al., 2010). Our results suggest that the FCM approach described here is also attractive to determine bacterial cell abundance in a range of other environments, especially when relative differences are evaluated, comparisons are made along broad gradients such as in our lake sediments, or OM contents are low. Coarse stream sediments and filter sludge, where OM contents tend to be lower than in many other environments, appear to be particularly well suited for FCM determinations of bacterial abundance. However, precise estimates of either method remain a challenge for samples high in OM content. Consequently, additional efforts will be needed to further improve protocols to count cells in such samples by either FCM or EM. One possible next step to test the accuracy of either method is to spike samples with fluorescent beads and determine recovery rates (Hammes and Egli, 2010).

Our protocol could also be expanded to quantify distinct taxonomic or functional groups of microbes in soils or other environments. The use of fluorescent *in situ* hybridization (FISH) techniques developed for EM (Bouvier and del Giorgio, 2003; Kubota, 2013) would seem particularly promising. Similarly, the abundance of cells differing in physiological state, such as fully active, dormant or dead (Lennon and Jones, 2011), could be determined after separating the cells from the soil or sediment matrix, and flow-cytometric (FCM) cell sorting is another powerful approach to distinguish specific microbes (Porter et al., 1993; Fawcett et al., 2011). Clearly, the combination of cell-separation, staining and FCM techniques holds large potential to enhance and greatly accelerate quantitative analyses of microbes in a range of environmental samples.

## Author Contributions

AF, MG, and FH designed the study, AF performed the sample analyses with the help of FH, AF analyzed data with the help of MG and AF and MG wrote the manuscript with significant inputs by FH.

## Conflict of Interest Statement

The authors declare that the research was conducted in the absence of any commercial or financial relationships that could be construed as a potential conflict of interest.
