# Supplementary material for: Flow Cytometric Assessment of Bacterial Abundance in Soils, Sediments and Sludge
Source: Front Microbiol. 2016 Jun 14;7:903. doi: 10.3389/fmicb.2016.00903 (PMC4905975; doi:10.3389/fmicb.2016.00903)
Supplement: Supplementary file 1 [file Table_1.PDF]

Supp. Table 1. Soil, sediment and sludge samples taken in a wide range of environments in Switzerland (i.e. stream and lake sediments, filter sludge, natural and agricultural soils). OM = organic matter

Sample type	Collection site	Collection date	GPS coordinates		%OM	Remarks	
Stream sediment	Val Roseg	11 Jun 2009	46°24'48" N	9°51'25" E	0.01	Glacier stream	Water temp. 3.1 °C
	Val Roseg	11 Jun 2009	46°24'45" N	9°51'15" E	0.02	Glacier stream	Water temp. 5.3 °C
	Val Roseg	11 Jun 2009	46°25'26" N	9°51'26" E	0.36	Glacier stream	Water temp. 6.4 °C
	Val Roseg	11 Jun 2009	46°25'13" N	9°51'41" E	2.54	Glacier stream	Water temp. 4.6 °C
	Val Roseg	11 Jun 2009	46°25'37" N	9°51'46" E	0.07	Glacier stream	Water temp. 8.4 °C
	Val Roseg	11 Jun 2009	46°25'51" N	9°51'52" E	0.87	Glacier stream	Water temp. 5.5 °C
	Sarine	12 Aug 2009	46°32'17" N	7°04'16" E	1.8	Lowland stream	Water temp. 17.2 °C
	Sarine	12 Aug 2009	46°32'20" N	7°04'17" E	1.42	Lowland stream	Water temp. 17.2 °C
	Sarine	12 Aug 2009	46°32'13" N	7°04'14" E	1.1	Lowland stream	Water temp. 13.3 °C
	Sarine	12 Aug 2009	46°32'8" N	7°05'18" E	0.87	Lowland stream	Water temp. 12.9 °C
	Sarine	12 Aug 2009	46°33'24" N	7°06'01" E	1.53	Lowland stream	Water temp. 16.1 °C
	Sarine	12 Aug 2009	46°34'6" N	7°05'41" E	2.61	Lowland stream	Water temp. 14.9 °C
Lake sediment	Klöntalersee	12 Apr 2011	47°01'30" N	8°58'27" E	4.44	Oligotrophic lake	Sampling depth 1 cm
	Klöntalersee	12 Apr 2011	47°01'30" N	8°58'27" E	4.14	Oligotrophic lake	Sampling depth 10 cm
	Klöntalersee	12 Apr 2011	47°01'30" N	8°58'27" E	3.12	Oligotrophic lake	Sampling depth 50 cm
	Lago di Comabbio	12 Apr 2011	45°45'41" N	8°41'32" E	32.97	Eutrophic lake	Sampling depth 1 cm
	Lago di Comabbio	12 Apr 2011	45°45'41" N	8°41'32" E	36.89	Eutrophic lake	Sampling depth 10 cm
	Lago di Comabbio	12 Apr 2011	45°45'41" N	8°41'32" E	22.86	Eutrophic lake	Sampling depth 50 cm
	Lago di Monate	12 Apr 2011	45°47'49" N	8°39'49" E	21.37	Meso-oligotrophic lake	Sampling depth 1 cm
	Lago di Monate	12 Apr 2011	45°47'49" N	8°39'49" E	16.44	Meso-oligotrophic lake	Sampling depth 10 cm
	Lago di Monate	12 Apr 2011	45°47'49" N	8°39'49" E	13.03	Meso-oligotrophic lake	Sampling depth 50 cm
	Lago di Endine	12 Apr 2011	45°46'47" N	9°56'23" E	7.93	Mesotrophic lake	Sampling depth 1 cm
	Lago di Endine	12 Apr 2011	45°46'47" N	9°56'23" E	7.45	Mesotrophic lake	Sampling depth 10 cm
	Lago di Endine	12 Apr 2011	45°46'47" N	9°56'23" E	5.18	Mesotrophic lake	Sampling depth 50 cm
	Englstensee	12 Apr 2011	46°46'26" N	8°21'23" E	4.58	Oligotrophic lake	Sampling depth 1 cm
	Englstensee	12 Apr 2011	46°46'26" N	8°21'23" E	4.83	Oligotrophic lake	Sampling depth 10 cm
	Englstensee	12 Apr 2011	46°46'26" N	8°21'23" E	2.29	Oligotrophic lake	Sampling depth 50 cm
Filter sludge	Drinking water plant Zurich	15 Dec 2010	47°22'36" N	8°32'30" E	0.69	Filter sludge	Filter age 22.7 yrs
	Drinking Water plant Zurich	15 Dec 2010	47°22'36" N	8°32'30" E	0.53	Filter sludge	Filter age 6.1 yrs
	Drinking Water plant Zurich	15 Dec 2010	47°22'36" N	8°32'30" E	0.72	Filter sludge	Filter age 7.3 yrs
	Drinking Water plant Zurich	15 Dec 2010	47°22'36" N	8°32'30" E	0.67	Filter sludge	Filter age 1.7 yrs
	Drinking Water plant Zurich	15 Dec 2010	47°22'36" N	8°32'30" E	0.51	Filter sludge	Filter age 20.7 yrs
	Drinking Water plant Zurich	15 Dec 2010	47°22'36" N	8°32'30" E	0.63	Filter sludge	Filter age 14.2 yrs
	Drinking Water plant Zurich	15 Dec 2010	47°22'36" N	8°32'30" E	0.51	Filter sludge	Filter age 4.9 yrs
	Drinking Water plant Zurich	15 Dec 2010	47°22'36" N	8°32'30" E	0.51	Filter sludge	Filter age 14.7 yrs
Natural Soil	Val Roseg	11 Jun 2009	46°24'48" N	9°51'25" E	0.09	Glacier forefield soil	
	Val Roseg	11 Jun 2009	46°24'45" N	9°51'15" E	0.91	Glacier forefield soil	
	Val Roseg	11 Jun 2009	46°25'26" N	9°51'26" E	2.39	Glacier forefield soil	
	Val Roseg	11 Jun 2009	46°25'13" N	9°51'41" E	0.19	Glacier forefield soil	
	Val Roseg	11 Jun 2009	46°25'37" N	9°51'46" E	0.29	Glacier forefield soil	
	Val Roseg	11 Jun 2009	46°25'51" N	9°51'52" E	0.63	Glacier forefield soil	
	Sarine floodplain	12 Aug 2009	46°32'19" N	7°04'18" E	1.23	Lowland soil	
	Sarine floodplain	12 Aug 2009	46°32'19" N	7°04'19" E	2.6	Lowland soil	
	Sarine floodplain	12 Aug 2009	46°32'13" N	7°04'14" E	5.33	Lowland soil	
	Sarine floodplain	12 Aug 2009	46°32'12" N	7°04'07" E	4.42	Lowland soil	
	Sarine floodplain	12 Aug 2009	46°32'10" N	7°04'06" E	7.75	Lowland soil	
	Sarine floodplain	12 Aug 2009	46°32'11" N	7°04'05" E	4.59	Lowland soil	
Agricultural soil	Trimmis	18 Aug 2009	46°53'58" N	9°33'52" E	4.39	Lowland agric. soil	CEC 14.5
	Diessenhofen	18 Aug 2009	47°41'22" N	8°45'01" E	2.98	Lowland agric. soil	CEC 10.7
	Siblingen	18 Aug 2009	47°42'45" N	8°31'26" E	6.81	Lowland agric. soil	CEC 26.7
	Oeschgen	18 Aug 2009	47°42'10" N	8°00'55" E	6.22	Lowland agric. soil	CEC 24.7
	Reckenholz	18 Aug 2009	47°25'44" N	8°31'09" E	7.92	Lowland agric. soil	CEC 39.1
	Mellikon	18 Aug 2009	47°34'06" N	8°21'05" E	2.77	Lowland agric. soil	CEC 11.8
	Gampelen	18 Aug 2009	47°00'53" N	7°03'31" E	43.59	Lowland agric. soil	CEC 101.3
	Gampelen-moor	18 Aug 2009	47°00'53" N	7°03'31" E	40.3	Lowland agric. soil	CEC 112.1
