# Supplementary material for: Flow Cytometric Assessment of Bacterial Abundance in Soils, Sediments and Sludge
Source: Front Microbiol. 2016 Jun 14;7:903. doi: 10.3389/fmicb.2016.00903 (PMC4905975; doi:10.3389/fmicb.2016.00903)
Supplement: Supplementary file 2 [file Table_2.PDF]

Supp. Table 2. Results of ANOVA and post-hoc tests showing differences in cell abundances g^{-1} dry mass, assessed with three different methods (FCM = flow cytometry, EM = epifluorescence microscopy, ATP = ATP quantification) for five different types of environments. DF = degrees of freedom (factor, residuals), F = F statistic. * indicates p<0.05.

Environment	Post-hoc test	DF	F	P
All environments		2, 164	34.4	<0.001*
	FCM vs. EM			*<0.001**
	FCM vs. ATP			*<0.001**
	EM vs. ATP			*0.001**
Stream sediment		2, 35	11.3	<0.001*
	FCM vs. EM			*<0.001**
	FCM vs. ATP			*0.036*
	EM vs. ATP			*0.096*
Lake sediment		2, 44	15.5	<0.001*
	FCM vs. EM			*0.581*
	FCM vs. ATP			*<0.001**
	EM vs. ATP			*<0.001**
Filter sludge		2, 23	24.1	<0.001*
	FCM vs. EM			*0.022**
	FCM vs. ATP			*0.002**
	EM vs. ATP			*<0.001**
Natural soil		2, 35	10.8	<0.001*
	FCM vs. EM			*0.001**
	FCM vs. ATP			*<0.001**
	EM vs. ATP			*0.989*
Agricultural soil		2, 23	30.2	<0.001*
	FCM vs. EM			*0.006**
	FCM vs. ATP			*<0.001**
	EM vs. ATP			*0.001**
